# Sociodemographic Comparison and Impact of Aila: The Supercyclone in Gosaba of West Bengal

**DOI:** 10.4103/0970-0218.69280

**Published:** 2010-07

**Authors:** Ranjan Bhattacharyya, Debasish Sanyal, Salil Kumar Dutta, Malati Ghosh, Sumita Bhattacharyya

**Affiliations:** Department of Psychiatry, Calcutta National Medical College, Kolkata India; 1 Department of Anthropology, Vidyasagar University, Midnapore, India; 2 Department of Pediatrics, Nil Ratan Sirkar Medical College, Kolkata, West Bengal, India

## Introduction

The South 24 Parganas district was one of the most affected areas of West Bengal when the recent cyclone Aila, which was a 250- to 350-km-wide beast with wind speeds exceeding 120 km/h, reached the east of India and Bangladesh on May 25, 2009. The cyclone affected an estimated 6.8 million people and left 138 individuals dead. Although, 18 of the 19 districts in West Bengal were affected, the situation was most precarious in South 24 Parganas and North 24 Parganas districts of the Sunderbans area. Sunderbans is one of the largest deltas of river Ganga with people of multiethnic background and pluralistic religious beliefs.(1) The people living here usually do not visit healthcare facility on its first opportunity and prefer to depend on religious faith-healers and local non-licensed village practitioners.(2) Gosaba, Basanti, Sagar, Patharpratima, and Namkhana were some of the most severely affected blocks. The Gosaba community development block consists of rural areas only with 14 *gram panchayats* (GPs). A huge unmet medical need in at-risk populations had been observed in the pos-tcyclone period as observed following cyclone Nargis and Rita.(3) Considering the stress, the present study had been conducted.

## Materials and Methods

This study was conducted in the four villages of Gosaba island: Rangabelia, Gosaba, Chhota Mollakhali and Bally II, which were selected on the basis of geographical clustering. The systematic random sampling taking every 20^th^ house had been done. A few severely damaged houses and unwilling family members had to be excluded. Individuals with intellectual or physical disability, those who were seriously ill were excluded from the study.

The following instruments were used in this study:

The *socio-economic status scale, rural (SES-rural)*: The SES status scale (rural) comprises nine items. The possible range of score varies from 0 to 54. The scale was originally devised by Pareek and Trivedi in 1964 and standardized in a village near New Delhi.(4)

*Trauma symptom checklist–40-item scale (TSC-40)*: The TSC-40 evaluates aspects of post-traumatic stress and other symptom clusters found in some traumatized individuals.(5) Each symptom item is rated according to its frequency of occurrence using a four-point scale ranging from 0 (never) to 3 (often). The TSC-40 also appears to predict the perpetration of intimate violence and war zone violence in women.(6)

*The clinician-administered dissociative states scale (CADSS)*: The scale measures dissociation in clinical observations at specific times. The scale measures 23 items in an interview.(7)

## Results

One hundred and sixty-six individuals, 40, 39, 42, and 45 from the Gosaba, Rangabelia, Bally II, and Chhota Mollakhali villages respectively, were interviewed. As shown in Table 1, there was no significant difference in the age of respondents across villages but a significant difference in terms of socio-economic status was found across the zones. The status was better in the main island (zone 1 Gosaba) than in the peripheral remote zones (zone 3 Bally II and zone 4 Chhota Mollakhali). The SES-rural total (SESr T) score also showed the mean SESr T score to be discrete (no overlapping) in regions of Bally II and Chhota Mollakhali. The most common variant of subitems of each individual item of the SES-rural score across the zones is shown in Table 2. The people of Gosaba primarily belong to dominant caste. They are high school educated, independent in occupation and live in *pucca* houses. Those of Chhota Mollakhali belong to lower caste, and majority of them are illiterate, earn their bread by cultivation, and live in *kuchcha* houses. The head-to-head comparison across the zones [Table 3] showed that the difference between every zone is significant at the level of 10^−3^ except between the zone 3 (Bally II) and zone 4 (Chhota Mollakhali) where the *P*-value is 0.046. The TSC score in the four zones as reflected by the chi-square test and mean rank is significantly higher in an ascending order in Gosba, Rangabelia, Bally II, and Chhota Mollakhali GPs [Table 4]. The TSC total mean score is lowest at the Gosaba GP and highest in the Chhota Mollakhali region [Table 5]. Applying Levene’s statistics, this difference is significant at the level of <0.001. After applying the Kruskal-Wallis test, the CADSS scale also showed significantly higher dissociative symptoms (*P*<0.001) in Bally II and Chhota Mollakhali regions than in other two regions [Table 6].

**Table 1:** Age and mean SES rural across the zones

Variables	*N*	Mean (95% CI)	SD	Significance (*P*-value)
Age zone				
1	40	30.65 (28.48–32.82)	6.800	
2	39	32.15 (29.62–34.69)	7.815	
3	42	32.43 (30.10–34.76)	7.487	0.326
4	45	30.00 (28.13–31.87)	6.223	
Total	166	31.28 (30.19–32.36)	7.093	
SESr T zone				
1	40	30.10 (27.39–32.81)	1.341	
2	39	27.85 (26.11–29.58)	0.857	
3	42	20.98 (18.66–23.29)	1.146	<0.001
4	45	17.40 (15.03–19.77)	1.176	
Total	166	23.82 (22.44–25.19)	0.697	

SD=standard deviation; CI=confidence interval.

**Table 2:** Highest frequencies of items of the SES-rural score across the zones

Variable	Zone 1 (Gosaba)	Zone 2 (Rangabelia)	Zone 3 (Bally II)	Zone 4 (Chhota Mollakhali)	Chi-square test, significance
Caste	6 (dominant) (*n*=14, 35%)	4 (agricultural) (*n*=10, 25.6%)	4 (agricultural) (*n*=15, 35.7%)	2 (lower caste) (*n*=17, 37.8%)	*χ*^2^=43.71, <0.001
Occupation	4 (independent) (*n*=21, 52.5%)	4 (independent) (*n*=11, 28.2%)	5 (cultivation) (*n*=15, 35.7%)	5 (cultivation) (*n*=14, 31.1%)	*χ*^2^=43.68, <0.001
Education	5 (high school) (*n*=16, 40.0%)	4 (middle) (*n*=13, 33.3%)	4 (middle) (*n*=11, 26.2%)	1 (illiterate) (*n*=18, 40.0%)	*χ*^2^=62.43, <0.001
Social participation	3 (office holder)(*n*=13, 32.5%)	3 (office holder) (*n*=12, 30.8%)	2 (member >1) (*n*=12, 28.6%)	1 (member <1) (*n*=17, 37.8%)	*χ*^2^=45.59, <0.001
Land	3 (5–10 acres) (*n*=11, 27.5%)	2 (1–5 acres) (*n*=17, 43.6%)	2 (1–5 acres) (*n*=14, 33.3%)	1 (<1 acre) (*n*=17, 37.8%)	*χ*^2^=67.66, <0.001
House	4 (pucca) (*n*=11, 27.5%)	3 (mixed) (*n*=16, 41.0%)	3 (mixed) (*n*=12, 28.6%)	2 (kutcha) (*n*=20, 44.4%)	*χ*^2^=87.69, <0.001
Farm power	0 (*n*o animal) (*n*=21, 52.5%)	0 (*n*o animal) (*n*=14, 35.9%)	2(1–2 animals) (*n*=21, 50.0%)	2 (1–2 animals) (*n*=22, 48.9%)	*χ*^2^=41.87, 0.001
Material possession	4 (chairs) (*n*=16, 40.0%)	3 (radio) (*n*=10, 25.6%)	2 (cycle) (*n*=17, 40.5%)	1 (bullock cart) (*n*=20, 44.4%)	*χ*^2^=74.99, <0.001
Family	1 (<5) (*n*=17, 42.5%)	1 (<5) (*n*=31, 79.5%)	1 (<5) (*n*=23, 54.8%)	2 (>5) (*n*=20, 44.4%)	*χ*^2^=37.58, <0.001

Digit codes and their interpretations are provided in parenthesess. Zone 1=Gosaba GP; Zone 2=Rangabelia GP; Zone 3=Bally II; Zone 4=Chota mollakhali

**Table 3:** Comparison between the TSC score total across the zones

Zone	*N*_1_, *N*_2_	Mean rank	Sum of ranks	Asymptotic sig. (two-tailed)	Monte carlo sig. (two-tailed)
Z_1_ and Z_2_	40, 39	Z_1_ 24.25 Z_2_ 30.56	944.00 1144.50	<0.001**	<0.001**
Z_2_ and Z_3_	39, 42	Z_2_ 31.86 Z_3_ 49.49	1242.50 2078.50	0.001**	0.001**
Z_3_ and Z_4_	42, 45	Z_3_ 38.32 Z_4_ 49.30	1609.50 2218.50	0.043*	0.046*
Z_4_ and Z_1_	45, 40	Z_4_ 60.56 Z_1_ 23.25	2725.00 930.00	<0.001**	<0.001**
Z_2_ and Z_4_	39, 45	Z_2_ 29.68 Z_4_ 53.61	1157.50 2412.50	<0.001**	<0.001
Z_1_ and Z_3_	40, 42	Z_1_ 24.65 Z_3_ 57.55	968.00 2417.00	<0.001	<0.001

* = *P*<0.05

** = *P*<0.01

**Table 4:** Trauma symptom checklist findings across zones

TSC score total	Zone	*N*	Mean rank	Chi-square	df	Asymptotic sig.	Monte carlo sig.
	1	40	34.54				
	2	39	74.22	71.980	3	<0.001	<0.001
	3	42	102.36				
	4	45	117.47				

TSC=trauma symptom checklist; df=degree of freedom

**Table 5:** Impact of trauma across the zones

Zone	*N*	TSC total mean (95% CI)	SD	df	Significance (Applying levene’s statistic)
Z_1_	40	6.90 (4.98–8.82)	5.99		
Z_2_	39	14.712 (12.51–16.93)	6.82	3	<0.001
Z_3_	42	20.81 (18.15–23.47)	8.54		
Z_4_	45	25.71 (22.33–29.09)	11.26		
Total	166	17.36 (15.67–19.04)	11.02		

Z_1_=Zone 1/Gosaba GP; Z_2_=Zone 2/Rangabelia GP; Z_3_=Zone 3/Bally II GP; Z_4_=Zone 4/Chota Mollakhali GP

**Table 6:** Dissociative symptoms across the zones

Zone	*N*	Mean (95% CI)	Std error	Mean rank	Sig. (Monte carlo)
Z_1_	40	4.15 (2.40–5.90)	0.864	34.99	
Z_2_	39	14.00 (10.47–17.53)	1.743	75.59	
Z_3_	42	19.12 (15.30–22.93)	1.889	95.54	<0.001
Z_4_	45	28.04 (24.45–31.64)	1.785	122.24	

CI=confidence interval

## Discussion

The present study showed that there is an increase in psychological symptoms following a natural calamity or man-made disaster.(8) The people having a low score in the SES-rural total score have a higher TSC total score and vice versa. The relief work and medical team reached relatively rapidly in Gosaba and Rangabelia and psychopathology was relatively less here. The people from Bally II and Chhota Mollakhali experienced more trauma-related memories as the severity of cyclone was more and relief was difficult to reach in these places. These findings replicate the findings of earlier studies that the impact of trauma is more in distal coastal areas than in the more proximal principal islands.(9) The PTSD is more common among males and unemployed individuals which has been replicated in this study.(10)

The attendance in GP camps with non-specific symptoms had increased to 34% in comparison to previous months before the cyclone in the Gosaba block which supports earlier findings.(11)

The scales used in the present studies are substantial in their merits and applications. The test-retest and interrater reliability of the SES-rural scale was found to be 0.87 and 0.93 respectively. The reliability of the TSC-40 scale and CADSS scale has also been assessed and Cronbach’s alpha value of these two scales found to be 0.93 and 0.90 respectively. Pearson’s correlation coefficient between the items of these two scales has been found to be 0.80 and the significance (two-tailed) is <0.01.

The previous studies using the TSC-40 indicate that it is a relatively reliable measure (subscale alphas typically range from 0.66 to 0.77, with alphas for the full scale averaging between 0.89 and 0.91).(12) The Cronbach-α for the total CADSS scale was found to be 0.73 in previous studies.(13)

The present study has several limitations. Only the Gosaba block and its surrounding villages had been surveyed. The sample size is only modest. The present study is only a cross-sectional survey. To avoid the interviewer bias, two researchers had collected data separately from each subject. The health status during the pre-cyclone period was not known so the comparison between the pre- and post-cyclone periods is only retrospective and recall bias cannot be ruled out.(14)

The impact of psychological trauma following cyclone Aila across the various zones of the Gosaba island has been reflected in this study. A larger community-based longitudinal study is perhaps needed to explore the long-term impact of the cyclone Aila.
